# Evaluating the Intensity of Exposure to MTV Shuga, an Edutainment Program for HIV Prevention: Cross-Sectional Study in Eastern Cape, South Africa

**DOI:** 10.2196/44111

**Published:** 2024-02-13

**Authors:** Sarah Mulwa, Venetia Baker, Cherie Cawood, David Khanyile, Dominique O'Donnell, Sophie Sarrassat, Simon Cousens, Isolde Birdthistle

**Affiliations:** 1 Faculty of Epidemiology & Population Health London School of Hygiene & Tropical Medicine London United Kingdom; 2 Epicentre Health Research Durban South Africa

**Keywords:** young people, media, evaluation, dose-response, edutainment, HIV prevention, mobile phone

## Abstract

**Background:**

MTV Shuga is an edutainment campaign designed to equip young people with knowledge, motivation, and informed choices to protect themselves from HIV infection. From 2019 to 2020, a total of 10 episodes of a new dramatic series, MTV Shuga “Down South 2” (DS2), were broadcast via television and the internet, alongside complementary media activities.

**Objective:**

This study aims to investigate whether the intensity of DS2 exposure was linked with positive HIV prevention outcomes in a setting with high HIV prevalence and relatively low levels of HIV testing.

**Methods:**

We analyzed data from a web-based survey of participants aged 15 to 24 years in South Africa in 2020. The survey was promoted via social media platforms of schools, universities, and communities in Eastern Cape, South Africa. The primary exposure of interest was the intensity of exposure to DS2, measured by the number of episodes of DS2 watched on the television or the internet or listened to on the radio (out of 10 episodes). Individuals who had not watched or listened to any DS2 episode were classified according to other MTV Shuga content that they had accessed. We estimated associations between the intensity of DS2 exposure and HIV-related outcomes, including knowledge of HIV status, awareness of HIV self-testing (HIVST) and pre-exposure prophylaxis (PrEP), uptake of HIVST, and demand for HIVST and PrEP, adjusting for potential confounders using multivariable logistic regression.

**Results:**

Among the 3431 survey participants, 827 (24.1%) were exposed to DS2. Specifically, 18.1% (622/3431) watched or listened to only 1 DS2 episode, and 2.4% (82/3431), 1.7% (58/3431), and 1.8% (62/3431) watched or listened to 2 to 4, 5 to 7, and 8 to 10 DS2 episodes, respectively. Increasing the exposure to DS2 was associated with improvements in most outcomes. Exposure to multiple episodes (eg, 2-4, 5-7, and 8-10) was associated with successively higher odds of knowing one’s HIV status, awareness of PrEP and HIVST, and uptake of HIVST compared with no MTV Shuga exposure, albeit with statistical uncertainty around some estimates. The interest in using HIVST or PrEP was high overall (>80%), with no measurable differences by DS2 intensity.

**Conclusions:**

We found evidence consistent with a dose-response relationship between MTV Shuga DS2 exposure and outcomes, including knowledge of HIV status, awareness and uptake of HIVST, and awareness of PrEP among young people in Eastern Cape. This indicates that greater engagement with a youth-focused edutainment campaign can improve HIV testing and prevention options in a setting and population with high need. However, only a few participants accessed multiple DS2 episodes despite its availability on multiple media platforms. We conclude that there is potential to benefit more young people by increasing access to and interest in the show.

## Introduction

### Background

Over the course of the HIV/AIDS epidemic, edutainment and mass media campaigns have played an important role in health promotion, with some successfully raising awareness of HIV transmission, prevention, and treatment as well as contributing to increases in condom use [1-10]. To reach their target audiences, campaigns have used different modes of delivery such as print (eg, comic books), audio (eg, radio), video campaigns (eg, television), celebrity influencers, the use of peers or community workers, or a combination of different strategies [1,10]. As technology and the internet have become more accessible, edutainment campaigns have expanded to incorporate digital solutions, such as social media, mobile phone apps, websites, and on-demand streaming channels, to achieve broader reach [3,11].

Progress in reducing new HIV infections has been reported in many countries [12]. However, gaps within the HIV prevention cascade persist among young people [13,14], a key target population for HIV prevention in many African countries. In South Africa, for instance, the analysis of survey data indicates that knowledge of HIV status among individuals aged 15 to 24 years has steadily increased from 19% in 2005 to 59% in 2017, but this proportion is still low compared with older age groups [15]. HIV testing is a crucial step in HIV prevention and treatment programming [12]. As a complement to provider- or client-initiated HIV testing and counseling, HIV self-testing (HIVST) can help reduce the testing gap among younger individuals who may prefer convenience and privacy and who may forego or be unable to access health care facilities [12,16]. In addition, awareness and uptake of pre-exposure prophylaxis (PrEP), which is now recommended as part of combination prevention for individuals at increased risk of HIV acquisition, remains low among young people [17]. As new HIV prevention options become available, edutainment campaigns can be adopted to enhance awareness through messaging that resonates with young people. This can, in turn, create demand for, facilitate uptake, and support the effective use of such options among young people.

Evidence of the impacts of edutainment campaigns that rely on dramatized series among young people has grown in recent years but remains limited [1,6,8,9]. MTV Shuga is an edutainment campaign designed to equip young people with knowledge and motivation to protect themselves from HIV infection while navigating healthy relationships. Shuga uses compelling characters and storylines to disseminate messages on HIV prevention through a series of parallel but interlinked storylines, which are offered over multiple episodes and media channels. From 2019 to 2020, a new series of MTV Shuga called Down South 2 (DS2), comprising 10 episodes, was produced in South Africa. DS2 episodes were broadcast via television and the internet alongside complementary media and offline activities, which included a documentary, peer-led discussions, DS2 graphic novels, and community-based viewing events [6,18]. The producers of DS2 conducted formative research through focus group discussions with young people to develop and validate the content, storylines, character development, and scripts, as they did for all previous MTV Shuga series [6,19,20]. Briefly, DS2 storylines largely revolve around characters who have recently left high school and are navigating life and the challenges that many young people face, including financial hardship, family conflict, and sexual relationships. Detailed descriptions of example storylines for DS2 have been documented previously [19]. In a recent evaluation of the DS2 series among young people aged 15 to 24 years in South Africa, we found that young people who engaged with the DS2 campaign (any component) were more likely to know their HIV status, use HIVST, and be aware of PrEP compared with those who did not engage with DS2 [6]. However, this analysis did not explore the intensity of DS2 exposure and whether greater exposure is linked with greater impacts.

In the absence of randomization, exploring whether the impact of an intervention differs across different intensities of exposure can support the plausibility of a causal association between the intervention and the outcomes [21,22]. Although little information exists regarding associations between the amount of exposure to edutainment campaigns and HIV-related outcomes among young people [1,8], published evidence from mass media campaigns in general populations suggests that high exposure intensity is associated with better health outcomes. In an evaluation of a weekly television soap opera on HIV/AIDS in Côte d’Ivoire by Shapiro et al [23], women who had watched ≥10 episodes (out of 20) were more likely to use condoms compared with nonviewers, whereas no effect was seen among women who watched <10 episodes. The authors concluded that repeated exposure to relevant content through continuous engagement partly contributed to the observed effects [23]. In an evaluation of an intervention to address domestic violence in South Africa (Soul City campaign, “SC4”), participants in rural residences with high exposure to SC4 television media (accessed ≥9 out of 13 episodes) were more likely to “do something to stop domestic violence” compared with those with no or low (<5 episodes) exposure, with little effect seen among those with moderate exposure (5-8 episodes) [24].

### Objectives

In this study, we investigated whether the intensity of DS2 exposure was linked with differential impacts on HIV prevention outcomes. Understanding and documenting such effects can offer insights to implementers and program designers on ways to maximize engagement and benefits for adolescents and young adults.

## Methods

### Study Setting, Sample, and Data Collection

The analysis for this paper was based on quantitative data from a mixed methods evaluation study conducted in 2020. This study aimed to evaluate the impacts of engagement with DS2 on HIV-related outcomes among young people in Eastern Cape, South Africa. The quantitative component used a self-administered web-based survey hosted on a website free of internet data charge for users and captured sociodemographic characteristics; exposure to MTV Shuga content (eg, how young people engage with DS2 and how many episodes they watched or listened to); and outcomes including knowledge of HIV status, awareness and uptake of HIVST, and awareness of PrEP. The survey was promoted via social media platforms; through targeted advertisements; and via social media accounts of schools, universities, and communities in the Eastern Cape. To avoid the risk of SARS-CoV-2 transmission, all study activities were conducted remotely. Additional details of the mixed methods evaluation are documented elsewhere [6].

### Measures

#### Exposure Variables

In this study, the primary exposure of interest was the intensity of exposure to DS2 dramatic series, measured by the number of episodes of DS2 watched on television or the internet or listened to on the radio (out of 10 episodes). Some individuals had not watched or listened to any DS2 episodes but accessed other formats of Shuga, including Down South 1 (DS1, the first series of Down South), which preceded DS2. Rather than grouping these individuals with those not exposed to any Shuga content, we classified them based on the specific MTV Shuga content they had accessed. These additional categories captured exposure to other MTV Shuga content, for which the intensity could not be inferred. On the basis of this definition, seven exposure categories are analyzed in this paper: (1) no exposure to MTV Shuga content, (2) exposure to any MTV Shuga format other than the DS2 dramatic series, (3) exposure to DS1 series, (4) exposure to only 1 DS2 episode, (5) exposure to 2 to 4 DS2 episodes, (6) exposure to 5 to 7 DS2 episodes, and (7) exposure to 8 to 10 DS2 episodes.

To understand with whom young people watched or listened to DS2 and whether this influenced the intensity of DS2 exposure and the impacts of exposure, we generated a secondary exposure variable with four categories as follows: watched or listened to DS2 (1) alone, (2) with peers only (eg, friends or partners), (3) with parents (eg, mother, father, grandparents and siblings or peers), and (4) with siblings (eg, siblings only or siblings and peers).

#### Outcome Variables

Outcomes included knowledge of HIV status, awareness and willingness to use HIVST and PrEP, and the uptake of HIVST. Furthermore, we analyzed 3 sexual behavior outcomes (ever had sex, had sex in the past 12 months, and condom use during last sex with current or last partner [in the past 12 months]). The definition of each of the outcomes is summarized in Multimedia Appendix 1. All measures were self-reported.

### Analysis

We summarized the frequencies and proportions of respondents reporting each of the above mentioned outcome measures based on the intensity of DS2 exposure. We estimated the associations between the intensity of DS2 exposure and each outcome using multivariable logistic regression models, adjusting for potential confounding variables. To limit the number of confounding variables adjusted for in the regression models, the selection of covariates in this study was informed by an earlier analysis that assessed the effects of binary exposure to DS2 (yes or no) on the abovementioned outcomes [6]. The initial set of confounding variables was identified using a directed acyclic graph to represent the hypothesized causal relationship between exposure to DS2, study outcomes, and other sociodemographic characteristics [6]. Only variables associated with each outcome at *P*≤.10 in the previous analysis were included in this analysis. From these regression models, we present the unadjusted and fully adjusted odds ratios (aORs) with their respective 95% CIs. To assess evidence of a dose-response relationship between DS2 intensity and the odds of each outcome, we compared 2 multivariable models: in the main model, the intensity of DS2 exposure was included as a categorical variable (model 1), whereas the other model assumed a linear relationship (model 0). The results from model 0 indicated whether the data were consistent with a linear trend, whereas a likelihood ratio test comparing model 0 with model 1 provided additional information regarding whether the relationship was more complex than linear [25]. All multivariable logistic regression models were restricted to individuals with nonmissing responses for each outcome. This decision was informed by the earlier analysis, which showed similar findings between complete case analysis and imputation methods [6]. In a secondary analysis, we assessed (1) whether the distribution of intensity of DS2 exposure varied by who young people watched or listened to DS2 with using *χ*^2^ tests and (2) whether these variations had differential effects on outcomes of interest following the regression approach described previously. This secondary analysis was conducted only among individuals who said they had watched or listened to DS2.

### Ethical Considerations

Ethics approvals were obtained from the Biomedical Research Ethics Committee at the University of KwaZulu-Natal (Ref: BREC/00000477/2019), the London School of Hygiene and Tropical Medicine (Ref: 17996), and the World Health Organization (Ref: ERC.0003283). All participants aged ≥18 years provided web-based consent, and participants aged <18 years provided their informed assent, with their parents or guardians providing informed consent [6]. To ensure participants’ confidentiality, we did not collect any identifying information other than mobile phone numbers for those who completed the survey to facilitate transfer of mobile data credit of ZAR50 (approximately US$5). We de-identified the data prior to conducting the analysis.

## Results

### Characteristics of the Study Sample

A total of 4145 records from the web-based survey were created by the users. In total, 82.8% (3431/4145) of the records were taken forward for analysis after removing records without full consent (407/4145, 9.8%) or sex information (144/4145, 3.5%) and likely duplicates (163/4145, 3.9%). We identified potential duplicates using a combination of date of birth and mobile phone numbers. Respondents were predominantly female (2020/3431, 58.9%), aged 20 to 24 years (2352/3431, 68.6% vs 1079/3431, 31.5% aged 15 to 19 years), enrolled in education (2857/3431, 83.3%), and resided in urban settings (2923/3431, 85.2%; Table 1). Household ownership of media assets was high, with proportions ranging from 56.8% (1949/3431) for computers or other digital devices to 82.5% (2832/3431) for televisions. Most respondents had their own smartphones (2922/3431, 85.2%), and 50.8% (1744/3431) had their own computer. Digital media engagement was high in the study population: 86.1% (2953/3431) reported using the internet and social media platforms at least once a week, and 74.1% (2542/3431) and 62.4% (2141/3431) watched television or listened to the radio at least weekly, respectively. A detailed summary of the study population has been described elsewhere [6].

**Table 1:** Sociodemographic characteristics of the survey participants overall and by intensity of DS2^a^ exposure (N=3431).

Characteristics		Exposure categories						
		No MTV Shuga exposure	Exposure to other MTV Shuga formats^b^	Exposure to DS1^c^	Exposure to 1 DS2 episode^d^	Exposure to 2-4 DS2 episodes	Exposure to 5-7 DS2 episodes	Exposure to 8-10 DS2 episodes
Total (N=3431), n (%)		1944 (56.7)	338 (9.9)	322 (9.4)	622 (18.1)	84 (2.5)	59 (1.7)	62 (1.8)
**Age group (y), n (%)**								
	15-19 (n=1079)	540 (50.1)	129 (12)	101 (9.4)	222 (20.6)	30 (2.8)	29 (2.7)	28 (2.6)
	20-24 (n=2352)	1404 (59.7)	209 (8.9)	221 (9.4)	400 (17)	54 (2.3)	30 (1.3)	34 (1.4)
**Sex, n (%)**								
	Male (1317)	851 (64.6)	99 (7.5)	101 (7.7)	199 (15.1)	34 (2.6)	19 (1.4)	14 (1.1)
	Female (n=2020)	1018 (50.4)	233 (11.5)	218 (10.8)	417 (20.6)	47 (2.3)	39 (1.9)	48 (2.4)
	Other (n=94)	75 (79.8)	6 (6.4)	3 (3.2)	6 (6.4)	3 (3.2)	1 (1.1)	0 (0)
**Schooling and employment, n (%)**								
	In school^e^ (n=726)	326 (44.9)	87 (12)	81 (11.2)	163 (22.5)	29 (4)	21 (2.9)	19 (2.6)
	TVET^f^ (n=967)	764 (79)	37 (3.8)	39 (4)	108 (11.2)	8 (0.8)	6 (0.6)	5 (0.5)
	University (n=1164)	513 (44.1)	139 (11.9)	139 (11.9)	272 (23.4)	36 (3.1)	29 (2.5)	36 (3.1)
	Employed (n=106)	57 (53.8)	11 (10.4)	14 (13.2)	18 (17)	6 (5.7)	0 (0)	0 (0)
	Unemployed (n=357)	212 (59.4)	47 (13.2)	42 (11.8)	47 (13.2)	4 (1.1)	3 (0.8)	2 (0.6)
	Unknown (n=111)	72 (64.9)	17 (15.3)	7 (6.3)	14 (12.6)	1 (0.9)	0 (0)	0 (0)
**Language spoken at home, n (%)**								
	English (n=259)	123 (47.5)	28 (10.8)	31 (12)	62 (23.9)	7 (2.7)	5 (1.9)	3 (1.2)
	IsiXhosa (n=2745)	1592 (58)	253 (9.2)	253 (9.2)	486 (17.7)	66 (2.4)	45 (1.6)	50 (1.8)
	Zulu (n=224)	106 (47.3)	31 (13.8)	21 (9.4)	46 (20.5)	7 (3.1)	5 (2.2)	8 (3.6)
	Other^g^ (n=203)	123 (60.6)	26 (12.8)	17 (8.4)	28 (13.8)	4 (2)	4 (2)	1 (0.5)
**Province, n (%)**								
	Eastern Cape-Mthatha (n=2462)	1472 (59.8)	196 (8)	204 (8.3)	452 (18.4)	51 (2.1)	40 (1.6)	47 (1.9)
	Eastern Cape-O.R. Tambo or other parts of Eastern Cape (n=364)	169 (46.4)	53 (14.6)	43 (11.8)	70 (19.2)	14 (3.8)	9 (2.5)	6 (1.6)
	Other provinces (n=536)	269 (50.2)	81 (15.1)	66 (12.3)	86 (16.0)	18 (3.4)	8 (1.5)	8 (1.5)
	Unknown (n=69)	34 (49.3)	8 (11.6)	9 (13.0)	14 (20.3)	1 (1.4)	2 (2.9)	1 (1.4)
**Residence, n (%)**								
	Urban setting (n=2923)	1665 (57)	276 (9.4)	277 (9.5)	535 (18.3)	70 (2.4)	47 (1.6)	53 (1.8)
	Rural setting (n=287)	133 (46.3)	37 (12.9)	34 (11.8)	57 (19.9)	9 (3.1)	10 (3.5)	7 (2.4)
	Unknown (n=221)	146 (66.1)	25 (11.3)	11 (5)	30 (13.6)	5 (2.3)	2 (0.9)	2 (0.9)
**Food insecurity, n (%)**								
	Never or rarely (n=1878)	1131 (60.2)	181 (9.6)	186 (9.9)	277 (14.8)	37 (2)	32 (1.7)	34 (1.8)
	Sometimes (n=1139)	576 (50.6)	118 (10.4)	114 (10)	261 (22.9)	30 (2.6)	21 (1.8)	19 (1.7)
	Often or always (n=218)	94 (43.1)	26 (11.9)	18 (8.3)	54 (24.8)	14 (6.4)	5 (2.3)	7 (3.2)
	Unknown (n=196)	143 (73)	13 (6.6)	4 (2)	30 (15.3)	3 (1.5)	1 (0.5)	2 (1)
**Relationship status, n (%)**								
	Not in a relationship (n=1453)	925 (63.7)	114 (7.8)	127 (8.7)	218 (15)	26 (1.8)	27 (1.9)	16 (1.1)
	Ever in a relationship (n=1317)	668 (50.7)	154 (11.7)	158 (12)	237 (18)	40 (3)	26 (2)	34 (2.6)
	Unknown (n=661)	351 (53.1)	70 (10.6)	37 (5.6)	167 (25.3)	18 (2.7)	6 (0.9)	12 (1.8)
**Ever had sex, n (%)**								
	No (n=1291)	1005 (77.8)	71 (5.5)	53 (4.1)	121 (9.4)	18 (1.4)	17 (1.3)	6 (0.5)
	Yes (n=1330)	462 (34.7)	187 (14.1)	228 (17.1)	324 (24.4)	48 (3.6)	37 (2.8)	44 (3.3)
	Unknown (n=810)	477 (58.9)	80 (9.9)	41 (5.1)	177 (21.9)	18 (2.2)	5 (0.6)	12 (1.5)
**Called a helpline or searched for information on HIV on the internet^h^, n (%)**								
	No (n=1667)	1172 (70.3)	113 (6.8)	86 (5.2)	204 (12.2)	41 (2.5)	28 (1.7)	23 (1.4)
	Yes (n=1352)	464 (34.3)	206 (15.2)	226 (16.7)	359 (26.6)	36 (2.7)	28 (2.1)	33 (2.4)
	Unknown (n=412)	308 (74.8)	19 (4.6)	10 (2.4)	59 (14.3)	7 (1.7)	3 (0.7)	6 (1.5)

^a^DS2: Down South 2.

^b^Formats other than the Down South dramatic series (eg, Alone Together miniseries on COVID-19).

^c^DS1: Down South 1.

^d^Individuals who had accessed DS2 formats not offered via radio, television, or the internet were classified as having watched 1 episode of DS2.

^e^Respondents in either primary or secondary school.

^f^TVET: technical and vocational education and training.

^g^Includes respondents whose language is unknown.

^h^This measure includes websites or helplines such as B-wise, Loveline, and Childline but excludes the MTV Shuga website.

### Intensity of Exposure to MTV Shuga DS2

The components and proportions comprising each exposure categories are summarized in Table 2. Of 3431 respondents, 1487 (43.3%) had been exposed to some form of MTV Shuga content. This comprised 9.9% (n=338) of the respondents exposed to MTV Shuga formats other than the DS2 dramatic series, 9.4% (n=322) of the respondents exposed to DS1 but not DS2, and 18.1% (n=622) of the respondents exposed to 1 DS2 episode. The proportion of respondents exposed to multiple DS2 episodes was low at approximately 2% within each category of 2 to 4 episodes (84/3431, 2.4%), 5 to 7 episodes (59/3431, 1.7%), or 8 to 10 episodes (62/3431, 1.8%). Repeated exposure to DS2 episodes was mainly through television or the internet (Table 2).

**Table 2:** Components used to define exposure categories and proportions of young people accessing each component (N=3431).

Exposure category and components		Values, n (%)
**No MTV Shuga exposure**		
	None	1944 (56.7)
**Exposure to other MTV Shuga formats^a^**		
	Watched the MTV preview of the show called 16 and Pregnant	856 (24.9)
	Watched MTV public service announcements with short MTV videos with health messages	596 (17.4)
	Watched any MTV Shuga Alone Together episodes on YouTube (Google LLC) or the MTV Shuga website	280 (8.2)
	Searched for information on HIV on the MTV Shuga website	210 (6.1)
	Answered a polling question about an MTV Shuga DS^b^ episode	267 (7.8)
	Ever posted any comments about an episode of MTV Shuga DS2^c^	294 (8.6)
	Exposed to other Shuga formats only: said yes to at least one of the above (but not exposed to DS1^d^ or DS2)	338 (9.9)
**Exposure to DS1 episodes^e^**		
	Ever watched MTV Shuga DS1 on television, MTV Shuga website, or YouTube	763 (22.2)
	Ever listened to MTV Shuga DS1 on the radio	245 (7.1)
	Exposed to DS1: said yes to at least one of the above (but not exposed to DS2)	322 (9.4)
**Exposure to 1 DS2 episode^f^**		
	Ever watched 1 MTV Shuga DS2 episode on television, MTV Shuga website, or YouTube	238 (6.9)
	Ever listened to 1 MTV Shuga DS2 episode on the radio	71 (2.1)
	Read the MTV Shuga DS2 graphic novel^g^	344 (10)
	Watched the documentary called MTV Shuga in real life that was broadcast at the end of DS2^g^	386 (11.3)
	Attended small group discussion facilitated by a peer educator on DS2 (at a clinic, school, university, TVET^h^, or somewhere else)^g^	513 (14.9)
	Attended a community event on DS2 anywhere^g^	292 (8.5)
	Exposed to 1 DS2 episode (exposure to any of the listed components)	622 (18.1)
**Exposure to 2-4 DS2 episodes^f^**		
	Ever watched 2-4 MTV Shuga DS2 episodes on television, MTV Shuga website, or YouTube	75 (2.2)
	Ever listened to 2-4 MTV Shuga DS2 episodes on the radio	30 (0.9)
	Exposed to 2-4 DS2 episodes either on television, MTV Shuga website, YouTube, or radio	84 (2.5)
**Exposure to 5-7 DS2 episodes^f^**		
	Ever watched 5-7 MTV Shuga DS2 episodes on television, MTV Shuga website, or YouTube	53 (1.5)
	Ever listened to 5-7 MTV Shuga DS2 episodes on the radio	17 (0.5)
	Exposed to 5-7 DS2 episodes either on television, MTV Shuga website, YouTube, or radio	59 (1.7)
**Exposure to 8-10 DS2 episodes^f^**		
	Ever watched 8-10 MTV Shuga DS2 episodes on television, MTV Shuga website, or YouTube	59 (1.7)
	Ever listened to 8-10 MTV Shuga DS2 episodes on the radio	7 (0.2)
	Exposed to 8-10 DS2 episodes either on television, MTV Shuga website, YouTube, or radio	62 (1.8)

^a^People exposed to other MTV Shuga formats only were not exposed to Down South 1 or Down South 2.

^b^DS: Down South series.

^c^DS2: Down South 2.

^d^DS1: Down South 1.

^e^Participants exposed to DS1 may have been exposed to other MTV Shuga formats (but not DS2).

^f^Participants exposed to DS2 may also have been exposed to DS1 or other MTV Shuga formats.

^g^Participants were classified as having watched 1 episode of DS2.

^h^TVET: technical and vocational education and training.

Of the 238 participants who had watched DS2 on television, MTV Shuga website, or YouTube, 162 (68.1%) had started watching DS2 the year before our evaluation. The intensity of exposure to DS2 was similar across most categories of participant characteristics, although there were some relatively small differences of approximately 5% to 8% in absolute terms for schooling or employment status, sexual history, and food insecurity: 9.7% (129/1330) of those who had ever had sex accessed ≥2 DS2 episodes compared with 3.2% (41/1291) of those who had never had sex, the corresponding proportions were 11.9% (26/218) among those who reported experiencing food insecurity often versus 6.2% (70/1139) among those who experienced moderate food insecurity, and approximately 9.5% (69/726) among those in school or university versus 2% (19/967) among those in technical and vocational education and training institutions (Table 1).

### Regression Results for DS2 Intensity

The proportion of respondents who knew their HIV status, were aware of PrEP and HIVST, and had used HIVST (ever and in the past year) increased with increasing DS2 exposure intensity. There was evidence of a nonlinear association between the intensity of DS2 exposure and knowledge of HIV status (nonlinear *P*=.06), awareness of PrEP (*P*<.001), and awareness of HIVST (*P*=.003). For uptake of HIVST and willingness to use HIVST or PrEP, the data were consistent with a linear trend (*P*=.38 for lifetime use of HIVST; *P*=.33 for use of HIVST in the past 12 months; *P*=.36 for willingness to test self with HIVST kit; *P*=.76 for willingness to give kit to partner; and *P*=.46 for willingness to take PrEP every day). The proportion of respondents reporting each of these outcomes was always the lowest among those not exposed to any MTV Shuga content (Multimedia Appendix 2).

**Figure 1:**
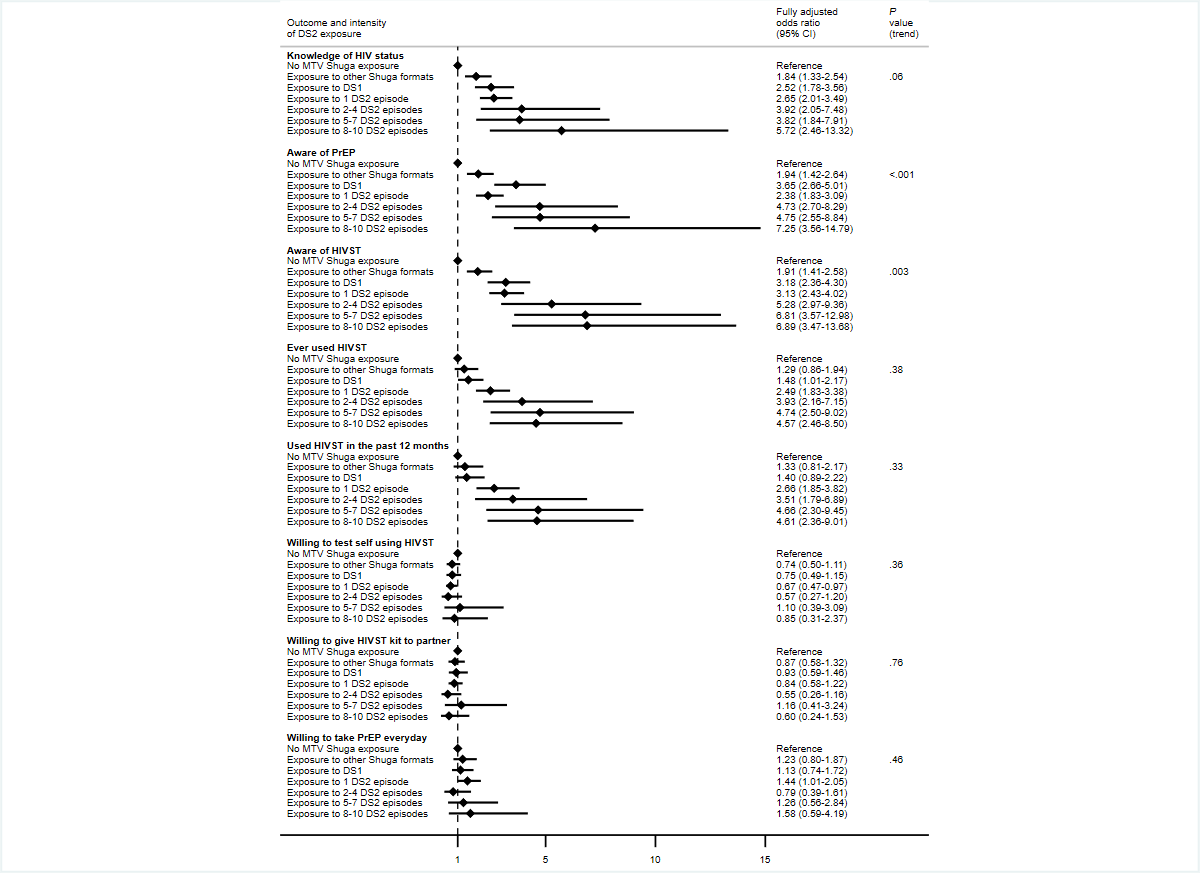
Forest plots summarizing the associations between different MTV Shuga Down South 2 (DS2) exposure intensities and HIV testing and pre-exposure prophylaxis (PrEP) outcomes. HIVST: HIV self-testing.

Knowledge of HIV status was 28.1% (431/1535) among those not exposed to any MTV Shuga content and highest among those who had watched or listened to 8 to 10 DS2 episodes (45/53, 84.9%; Multimedia Appendix 2). In the adjusted analysis, increasing DS2 exposure was associated with increasing knowledge of HIV status. Compared with individuals not exposed to any MTV Shuga content, the aORs for those who had watched or listened to 1 DS2 episode, 2 to 4 DS2 episodes, 5 to 7 DS2 episodes, and 8 to 10 DS2 episodes were 2.65 (95% CI 2.01-3.49), 3.92 (95% CI 2.05-7.48), 3.82 (95% CI 1.84-7.91), and 5.72 (95% CI 2.46-13.32), respectively (Figure 1; Multimedia Appendix 2).

Similarly, increasing DS2 exposure was associated with increased awareness of PrEP in the adjusted analysis: for example, 47.2% (215/456) of those who had watched or listened to only 1 DS2 episode versus 17.1% (251/1469) of those not exposed to any MTV Shuga content (aOR 2.38, 95% CI 1.83-3.09) and 76% (38/50) of those who had watched or listened to 8 to 10 episodes versus 17.1% (251/1469) of those who were not exposed to any MTV Shuga content (aOR 7.25, 95% CI 3.56-14.79; Figure 1; Multimedia Appendix 2). Exposure to DS2 was not associated with willingness to take PrEP (which was high overall at 1851/2284, 81%; Figure 1; Multimedia Appendix 2).

Regarding lifetime awareness of HIVST (ever heard of HIVST), proportions ranged from 18.8% (284/1509) among those who were not exposed to any MTV Shuga content to 56.4% (270/479) among those who had watched or listened to only 1 DS2 episode and to 75% (39/52) among those who had watched or listened to 8 to 10 episodes. In the adjusted analysis, those exposed to 2 to 4, 5 to 7, or 8 to 10 episodes of DS2 had >5 times higher odds of being aware of HIVST compared with those not exposed to any MTV Shuga content (Figure 1; Multimedia Appendix 2). Lifetime use of HIVST was 26.4% (125/474) among those who had watched or listened to only 1 DS2 episode versus 7.8% (115/1483) among those not exposed to any MTV Shuga content (aOR 2.49, 95% CI 1.83-3.38), 32.8% (21/64) among those who had watched or listened to 2 to 4 DS2 episodes (aOR 3.93, 95% CI 2.16-7.15), 35.2% (19/54) among those who had watched or listened to 5 to 7 episodes (aOR 4.74, 95% CI 2.50-9.02), and 40% (21/53) among those who watched or listened to 8 to 10 episodes (aOR 4.57, 95% CI 2.46-8.50).

Likewise, in the adjusted analysis, watching or listening to an increasing number of DS2 episodes was associated with increased odds of using HIVST in the past year (Figure 1; Multimedia Appendix 2). Among those who had never used an HIVST before, interest in using HIVST on oneself or interest in giving an HIVST kit to a partner was high overall (83% for both), with no differences in the number of DS2 episodes accessed (Figure 1; Multimedia Appendix 2).

**Figure 2:**
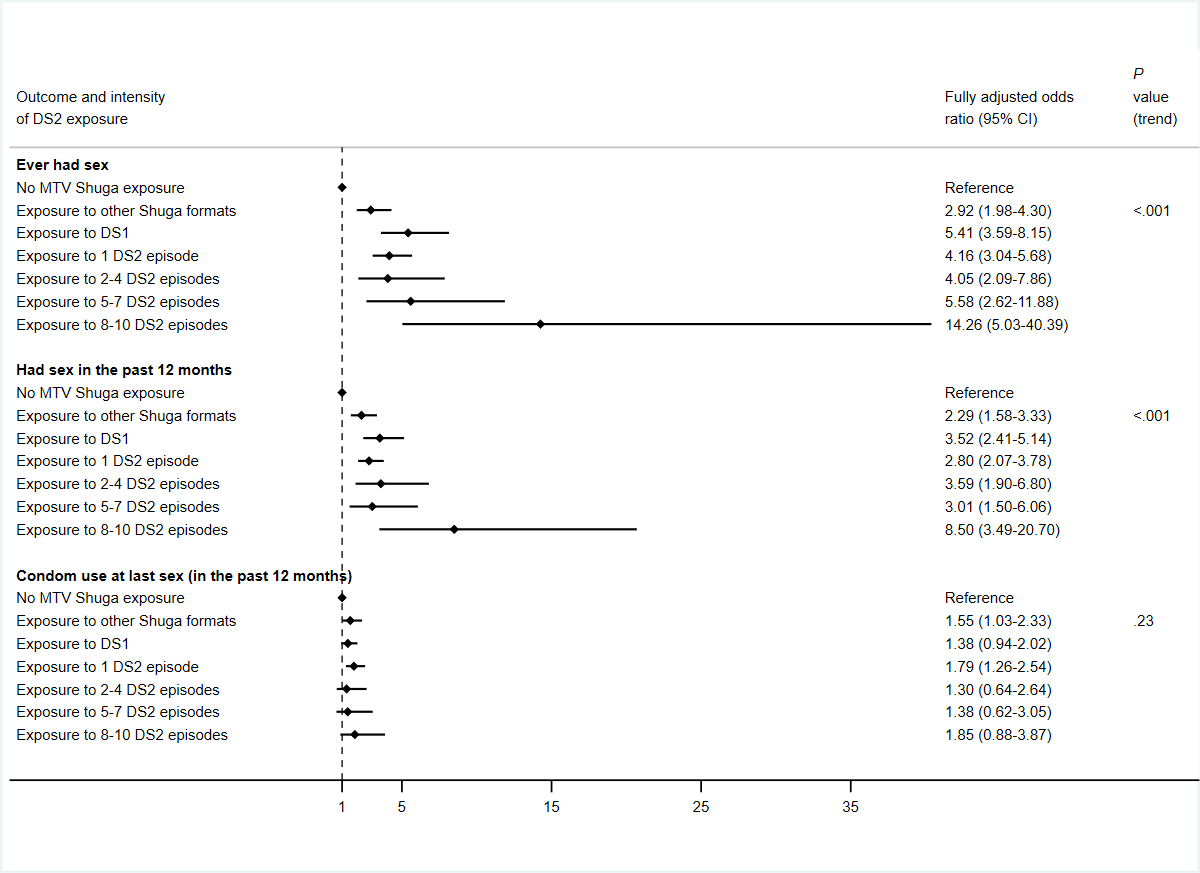
Forest plots summarizing the associations between different MTV Shuga Down South 2 (DS2) exposure intensities and sexual behavior outcomes.

Compared with respondents who were not exposed to any MTV Shuga content, DS2 audiences who had watched or listened to multiple episodes of DS2 were more likely to ever had sex in their lifetime and had sex in the past 12 months (Figure 2; Multimedia Appendix 3). Among those who reported having sex within the past 12 months, there was no evidence of a departure from a linear trend between DS2 exposure intensity and condom use (*P*=.23; Figure 2; Multimedia Appendix 3). There was evidence of a nonlinear association between DS2 exposure intensity and sexual history (ever or in the past 12 months; nonlinear *P*<.001).

### Effects of Other MTV Shuga Content or Formats

Regarding knowledge of HIV status, awareness of PrEP and HIVST, and lifetime uptake of HIVST, there was evidence that exposure to other forms of MTV Shuga content (other than the DS2 dramatic series) was beneficial, compared with no exposure to MTV Shuga content. For instance, knowledge of HIV status was 74.4% (215/289; aOR 2.52, 95% CI 1.78-3.56) among those who had watched DS1 and 63% (182/289; aOR 1.84, 95% CI 1.33-2.54) among those who had accessed other MTV formats compared with 28.1% (431/1535) among those with no exposure to MTV Shuga content (Figure 1; Multimedia Appendix 2). Furthermore, those exposed to these other forms of MTV Shuga content were more likely to have had sex (ever or within the past 12 months) compared with those with no MTV Shuga exposure (Figure 2; Multimedia Appendix 3).

### How Young People Watched or Listened to DS2

**Figure 3:**
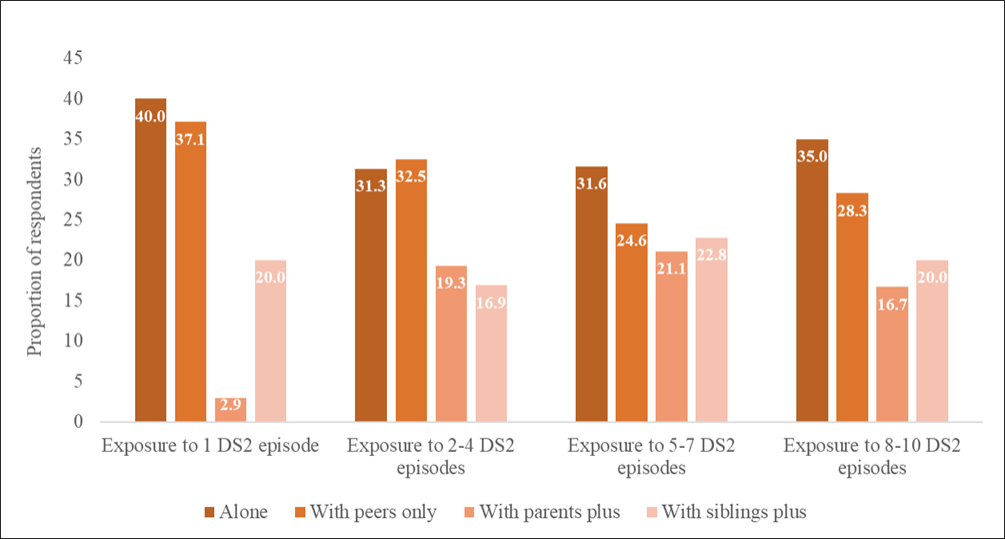
Intensity of Down South 2 (DS2) exposure by how young people watched or listened to DS2 episodes.

Among those who had been exposed to at least 1 DS2 episode and provided information on how they accessed the series, 50.6% (119/235) had watched or listened to DS2 with someone, 33.6% (79/235) accessed alone, and 15.7% (37/235) reported a combination of the 2 (Table 3). Among those who had watched or listened to DS2 with someone, 45.5% (71/156) did so with peers only, 25% (39/156) with parents, and 29.5% (46/156) with siblings. The findings were similar by age group (*P*=.45; Table 3). The intensity of DS2 exposure did not differ significantly by how young people accessed DS2 (*P*=.46), although higher proportions of repeat viewers watched or listened to DS2 with a parent than those who watched or listened to 1 DS2 episode. For example, 19.3% (16/83) of the participants who accessed 2 to 4 DS2 episodes did so with a parent compared with 2.9% (1/35) among those who accessed 1 DS2 episode (Figure 3). We were unable to establish whether our outcomes of interest differed by the intensity of DS2 exposure, given how young people accessed DS2, because of insufficient data to conduct the regression analyses.

**Table 3:** Descriptive summaries of how young people watched or listened to Down South 2.

Question and response			Age group (y), n (%)					Total (n=235), n (%)^a^
			15-19 (n=104)^a^		20-24 (n=131)^a^			
**Did you usually watch or listen to MTV Shuga Down South season 2 on your own or with someone?**								
	On my own		31 (29.8)		48 (36.6)		79 (33.6)	
	With someone		54 (51.9)		65 (49.6)		119 (50.6)	
	Both		19 (18.3)		18 (13.7)		37 (15.7)	
**With whom did you usually watch or listen to MTV Shuga Down South season 2?**								
	Alone		31 (29.8)		48 (36.6)		79 (33.6)	
	**With peers only**		27 (26)		44 (33.6)		71 (30.2)	
		Friends	24 (32.9)	30 (36.1)		54 (34.6)		
		Friends and partners	1 (1.4)	9 (10.8)		10 (6.4)		
		Partners	2 (2.7)	5 (6)		7 (4.5)		
	**With parents plus**		22 (21.2)		17 (13)		39 (16.6)	
		Parents	6 (8.2)	3 (3.6)		9 (5.8)		
		Parents and friends	4 (5.5)	1 (1.2)		5 (3.2)		
		Parents, friends, and partners	1 (1.4)	1 (1.2)		2 (1.3)		
		Parents and siblings	5 (6.8)	3 (3.6)		8 (5.1)		
		Parents, friends, and siblings	4 (5.5)	2 (2.4)		6 (3.8)		
		Parents, friends, partners, and siblings	2 (2.7)	6 (7.2)		8 (5.1)		
		Parents, partners, and siblings	0 (0)	1 (1.2)		1 (0.6)		
	**With siblings plus**		24 (23.1)		22 (16.8)		46 (19.6)	
		Siblings	15 (20.5)	11 (13.3)		26 (16.7)		
		Siblings and friends	7 (9.6)	6 (7.2)		13 (8.3)		
		Siblings, friends, and partners	2 (2.7)	2 (2.4)		4 (2.6)		
		Siblings and partners	0 (0)	3 (3.6)		3 (1.9)		

^a^Among those who had watched or listened to at least 1 Down South 2 episode and provided information on how they accessed the episodes.

## Discussion

### Key Findings

This study aimed to understand whether the intensity of exposure to the MTV Shuga DS2 series had differential impacts on the outcomes of interest. We found evidence that the levels of knowledge of HIV status, awareness of PrEP, and use of HIVST (ever or in the past year) increased with increased exposure to DS2, consistent with a dose-response effect. We found no evidence of an effect of DS2 exposure intensity on the interest in using HIVST or PrEP (a proxy for demand), which was already high in the study population. In our study sample, repeated exposure to DS2 episodes was mainly through television or the internet.

Regarding how young people access DS2, findings indicate a mix of preferences, with the majority watching or listening to DS2 alone or with peers (eg, friends or partners) and less so with siblings and parents. Although there was little influence of these preferences on the intensity of DS2 exposure, we found higher proportions of repeat viewers watched or listened to DS2 with a parent, compared with those who accessed only 1 episode. This suggests that accessing DS2 with parents may have helped some young people to watch more episodes. However, it is worth noting that some of our study participants avoided watching with parents because they feared that it would be awkward or that the parents would judge or lecture them on sex and relationship matters, as documented in our related qualitative research [19]. Others may have preferred watching or listening to DS2 alone for privacy reasons, as documented in another study [11]. These findings point toward the need to ensure privacy and nonjudgmental spaces in the delivery of campaigns targeted to young populations. Our related qualitative research showed that watching or listening to DS2 spurred interpersonal conversations and discussions with peers and sometimes with parents, as reported elsewhere [3,19]. Discussions where peers engaged in debates about DS2 characters and storylines led to shared learning and support systems where people felt safe to discuss sexual health topics and disclose personal information [19]. This could deepen the influence of the show for the viewer (beyond just watching), for example, through greater internalization, and future research could consider these events as mediators along a pathway between exposure (eg, to MTV Shuga content) and HIV and health outcomes.

The observed dose-response effects on knowledge of HIV status, awareness of PrEP, and use of HIVST (ever or in the past year) might reflect that more exposure facilitates increased levels of narrative engagement and, in turn, helps the viewers or listeners to retain a greater amount of relevant content compared with someone with little or no exposure. This might particularly be true when the audience is interested in finding out what eventually happens to the characters, often facilitated by the immersive nature of the DS2 series. As documented in our qualitative research, the emotional and dramatic storylines kept participants engaged as they wanted to know what would happen next [19]. In addition, the re-emphasis of content across multiple storylines allows participants to access content that they may have missed previously. Previous research has documented that participants in dramatized series tend to actively reflect on the plotlines and often compare the characters’ ways of confronting dilemmas across storylines, which presents a learning opportunity for the audience [3,5]. In a cluster randomized trial of MTV Shuga in Nigeria, the effects of MTV Shuga were stronger for viewers who were more immersed or highly identified with the characters while watching the show [9]. Although we did not measure identification with characters in this analysis, we drew on other evidence from the larger mixed methods study, which indicates that young people found the show relatable. In a miniseries called *MTV Shuga: Alone Together* (which aimed to disseminate timely and accurate information on COVID-19), developed in the same manner as DS2, ≥90% of young people (in the same study sample as this paper) who had watched the miniseries indicated that they found it entertaining, informative, memorable, and realistic (I Birdthistle, PhD, unpublished data, February 2021). One of the key findings from the nested qualitative study conducted as part of our research to understand why young people engaged with DS2 was that young people found the show relatable as the storylines reflected real-life issues that they or people they knew experienced [19]. Younger participants appreciated how the show embraced the uncertainty that often surrounds such decision-making (as opposed to simplistic or moralistic messaging). In addition, some viewers reported being introduced to HIVST and PrEP for the first time through the show, and less experienced viewers felt more prepared for future sexual relationships and decision-making based on the scenarios explored in DS2 [19]. The high identification with the DS2 characters and storylines likely enhanced the effect of watching DS2. The larger effects among those who watched or listened to multiple DS2 episodes suggest the potential benefits of viewing dramas as a whole rather than as a series of parallel storylines, as documented elsewhere [5]. However, it is worth noting that a viewer or listener may miss relevant content completely when scenes are relatively few and short [6]. Although there is uncertainty around our effect estimates, given the low proportion of participants who accessed multiple episodes of DS2, there remains a clear overall pattern for these outcomes (except those on demand) that more DS2 exposure is better.

We did not find evidence of a dose-response relationship between DS2 exposure intensity and demand outcomes (ie, interest in using HIVST and PrEP), which were already high in the study population. Similarly, higher DS2 exposure intensity did not result in greater condom use. Although DS2 influenced awareness and motivation to use HIV prevention products and services, including HIV testing and PrEP, the actual provision of these products was not part of the DS2 campaign [6]. Qualitative research findings indicate that many participants were unsure of the availability of these services and products in their own setting, which could partly explain the limited effect on demand for these products [6]. This finding highlights the crucial role that actual provision and access to these HIV prevention tools play in influencing behavior, in addition to awareness and motivation to use these methods [26]. It is possible that our analysis is underpowered to detect smaller differences when assessing the dose-response effect for demand measures, given the high absolute proportions of these outcomes in our study sample and the fact that very few study participants accessed multiple DS2 episodes. MTV Shuga audiences who had watched or listened to multiple episodes were more likely to have had sex in their lifetime or in the past 12 months compared with those who had never engaged with the campaign, suggesting that DS2 may be reaching those most in need of HIV prevention methods. In addition, given the social desirability associated with reporting of sexual behaviors, it is possible that those who watched MTV Shuga episodes felt less judged or stigmatized and so were more likely to be open about sexual history.

Only 6% (205/3431) of the study respondents had accessed ≥2 DS2 episodes despite their availability on multiple media platforms (eg, YouTube [Google LLC], South African Broadcasting Corporation, local radio stations, and MTV Shuga website) and our sample’s high engagement with media (3101/3431, 90.4% watching television, listening to the radio, or watching YouTube at least once a week). The low repeated exposure in our study is consistent with results from an evaluation of MTV Shuga DS1 in KwaZulu-Natal, where only 8% of 1853 adolescent girls and young women aged 13 to 23 years had watched ≥1 episode (out of 24 episodes) [27]. In an evaluation of a 24-episode drama series on sexual and reproductive health among Latinx adolescents and young women in the United States in which participants were randomized to either a control group or to 1 of the 4 experimental arms (based on different storytelling formats), only 6% of those assigned to experimental arms went on to watch the full episodes after exposure to different formats [3]. These estimates are much lower than those from multimedia studies targeting general populations, where exposure to multiple episodes (≥5) is as high as 30% [4,23].

The low exposure to multiple episodes in our study may reflect various issues inherent in engaging a relatively young population (aged <25 years). First, young viewers or listeners often have no control over when episodes are aired on television or radio channels and given that they might actively avoid watching or listening to MTV Shuga episodes with parents or older siblings for reasons summarized previously, this can limit their ability to access complete or multiple episodes. Limited affordability and availability of the internet and data plans may prohibit access to content offered via streaming platforms such as YouTube or the MTV Shuga website [28]. Even with extensive efforts to create relatable storylines, we acknowledge that it can be challenging to capture the wide range of complexities in the lives of adolescents and young adults in 1 show, and there may be a proportion of participants who are not interested in the show entirely. In addition, it is possible that some young people may have decided to stop watching or listening after 1 episode because they did not find the episode interesting enough. Future research can aim to understand the complete range of reasons for “disengagement” in different contexts and age groups.

Given our study findings that more DS2 exposure is better for many of the outcomes and the fact that a high proportion of our study sample engaged with “offline” DS2 content, it might be worth finding ways to facilitate and increase offline viewing of DS2 episodes. In particular, peer education and school education programs are good complementary options because they (1) do not require constant internet connectivity (which may prohibit access via YouTube or MTV Shuga website) and (2) can create a safe space for young people (who prefer to watch alone or with peers) to watch without having to worry about the reactions and judgment of older adults. In addition, short clips or extensions can be used to deliver and highlight critical components (eg, HIV testing and PrEP) so that people do not miss them. These could be offered through social media platforms, which are popular among young people. We note here that the specific combinations of activities that program designers and implementers use will depend on the scope and goals of the campaign. For instance, if promoting high intensity of exposure (ie, access to multiple episodes) is the goal, then complementary activities may need to incorporate and deliver full episodes rather than sharing shorter formats (eg, on social media), with the latter more suited for raising awareness and enhancing interest in general. Many web-based platforms also allow viewers to download episodes and watch or listen later, and sensitizing young people about this option can potentially facilitate more access. The recent inclusion of MTV Shuga on Netflix is likely to widen its reach, although it may reach those who are already connected and able to pay for the service. Furthermore, for some young people, getting their parents interested in watching Shuga might improve their exposure intensity. As we learn from the COVID-19 pandemic, public health players need to be innovative and adaptive, and using a combination of strategies to reach the target populations is worthwhile [29].

### Strengths and Limitations

One of the study limitations is that we relied on self-reported measures that are subject to recall bias. Some of the behavioral questions (eg, on sexual history) may also be subject to social desirability bias. To ensure participants’ confidentiality and to help minimize social desirability bias, we did not collect any identifying information (although mobile phone numbers for those who completed the survey were obtained to facilitate the transfer of mobile data credit). Furthermore, the survey was self-administered, and we anticipated that participants would complete the interviews in private, which could increase the accuracy of the self-reported information. Regarding recall bias, we feel that most of our behavioral outcome measures are related to events that people are likely to remember (eg, testing for HIV in the last year and taking PrEP). Many of our questions included options such as “don’t know” and “prefer not to answer” to accommodate participants who may not have an opinion regarding the question at hand. Approximately 6 (68%) out of 10 participants had started watching DS2 the year before we conducted our evaluation, and this may have influenced their ability to accurately recall the exact number of DS2 episodes accessed. Participants who were exposed to DS2 but did not know how many episodes they had watched or listened to were assumed to have been exposed to only 1 DS2 episode. Furthermore, we did not collect information on how frequently offline components such as graphic novels were accessed, and thus, individuals exposed to DS2 content not offered through radio, television, or the internet were also assumed to have been exposed to only 1 DS2 episode. We may have misclassified these individuals if they had indeed been exposed to multiple episodes. Although we adjusted for various potential confounders in all our analyses, we cannot rule out residual confounding and other possible explanations for the observed associations. Moreover, it is possible that those with a higher awareness of HIV in general might be more likely interested in accessing DS2, resulting in reverse causality. The strengths of the study include the assessment of multiple outcomes, the rich data on exposure to multiple MTV Shuga content, and information on how young people accessed DS2. Among those not exposed to DS2, we were able to capture exposure to other MTV Shuga content. Although this resulted in “nonnatural” categories of DS2 exposure intensity, it allowed us to identify whether individuals exposed to other MTV Shuga content had better outcomes than those not exposed to any MTV Shuga content at all.

### Conclusions

Several studies have examined the effects of edutainment campaigns on sexual and HIV-related health outcomes among young people; however, few have examined the intensity of exposure and whether increased engagement resulted in greater benefits. In this study, we found that increasing DS2 exposure was associated with increasing knowledge of HIV status, awareness of PrEP, and use of HIVST. This is consistent with a dose-response effect and supports the plausibility of a causal association between DS2 and HIV prevention outcomes among young audiences. Overall, relatively few participants viewed multiple episodes of DS2, and supporting young people to view or listen to more episodes of the DS2 campaign can yield benefits for more young people. If promoting high intensity of DS2 exposure (ie, access to multiple episodes) is the goal for a given campaign, incorporating complementary activities that support the delivery of full episodes rather than sharing shorter formats may be useful. A more complete and immersive experience can be offered through better and more equal digital access and through school programs and peer education programs, taking into account young people’s preferences when designing and delivering these campaigns.

## Appendix

Multimedia Appendix 1 Definitions of outcome measures included in the analysis.

Multimedia Appendix 2 Associations between different MTV Shuga Down South 2 exposure intensities and HIV testing and pre-exposure prophylaxis outcomes.

Multimedia Appendix 3 Associations between different MTV Shuga Down South 2 exposure intensities and sexual behavior outcomes.

## Abbreviations

aOR: adjusted odds ratio
DS1: Down South 1
DS2: Down South 2
HIVST: HIV self-testing
PrEP: pre-exposure prophylaxis

## Data Availability

The data sets generated and analyzed during this study are available from the corresponding author upon reasonable request.

## References

[1] LaCroix JM, Snyder LB, Huedo-Medina TB, Johnson BT (2014). Effectiveness of mass media interventions for HIV prevention, 1986-2013: a meta-analysis. J Acquir Immune Defic Syndr.

[2] Jones R, Hoover DR, Lacroix LJ (2013). A randomized controlled trial of soap opera videos streamed to smartphones to reduce risk of sexually transmitted human immunodeficiency virus (HIV) in young urban African American women. Nurs Outlook.

[3] Wang H, Singhal A (2016). East Los High: transmedia edutainment to promote the sexual and reproductive health of young Latina/o Americans. Am J Public Health.

[4] Do MP, Kincaid DL (2006). Impact of an entertainment-education television drama on health knowledge and behavior in Bangladesh: an application of propensity score matching. J Health Commun.

[5] Lovell CC, Pappas-Deluca KA, Sebert Kuhlmann AK, Koppenhaver T, Kong S, Mooki M, Galavotti C (2007). "One day I might find myself HIV-positive like her": audience involvement and identification with role models in an entertainment-education radio drama in Botswana. Int Q Community Health Educ.

[6] Birdthistle I, Mulwa S, Sarrassat S, Baker V, Khanyile D, O'Donnell D, Cawood C, Cousens S (2022). Effects of a multimedia campaign on HIV self-testing and PrEP outcomes among young people in South Africa: a mixed-methods impact evaluation of 'MTV Shuga Down South'. BMJ Glob Health.

[7] Shilubane T, Geyer S (2014). khomanani: an HIV and AIDS community mobilisation programme for resource-constrained settings. Social Work.

[8] Orozco-Olvera V, Shen F, Cluver L (2019). The effectiveness of using entertainment education narratives to promote safer sexual behaviors of youth: a meta-analysis, 1985-2017. PLoS One.

[9] Banerjee A, La Ferrara EL, Orozco-Olvera VH (2019). The entertaining way to behavioral change: fighting HIV with MTV. National Bureau of Economic Research.

[10] Taggart T, Ritchwood TD, Nyhan K, Ransome Y (2021). Messaging matters: achieving equity in the HIV response through public health communication. Lancet HIV.

[11] Jones R, Lacroix LJ (2012). Streaming weekly soap opera video episodes to smartphones in a randomized controlled trial to reduce HIV risk in young urban African American/black women. AIDS Behav.

[12] UNAIDS (2022). In danger: UNAIDS global AIDS update 2022. Joint United Nations Programme on HIV/AIDS (UNAIDS).

[13] Hayes RJ, Donnell D, Floyd S, Mandla N, Bwalya J, Sabapathy K, Yang B, Phiri M, Schaap A, Eshleman SH, Piwowar-Manning E, Kosloff B, James A, Skalland T, Wilson E, Emel L, Macleod D, Dunbar R, Simwinga M, Makola N, Bond V, Hoddinott G, Moore A, Griffith S, Deshmane Sista N, Vermund SH, El-Sadr W, Burns DN, Hargreaves JR, Hauck K, Fraser C, Shanaube K, Bock P, Beyers N, Ayles H, Fidler S (2019). Effect of universal testing and treatment on HIV incidence - HPTN 071 (PopART). N Engl J Med.

[14] Floyd S, Shanaube K, Yang B, Schaap A, Griffith S, Phiri M, Macleod D, Sloot R, Sabapathy K, Bond V, Bock P, Ayles H, Fidler S, Hayes R (2020). HIV testing and treatment coverage achieved after 4 years across 14 urban and peri-urban communities in Zambia and South Africa: an analysis of findings from the HPTN 071 (PopART) trial. PLoS Med.

[15] Jooste S, Mabaso M, Taylor M, North A, Tadokera R, Simbayi L (2020). Trends and determinants of ever having tested for HIV among youth and adults in South Africa from 2005-2017: results from four repeated cross-sectional nationally representative household-based HIV prevalence, incidence, and behaviour surveys. PLoS One.

[16] Inwani I, Chhun N, Agot K, Cleland CM, Rao SO, Nduati R, Kinuthia J, Kurth AE (2021). Preferred HIV testing modalities among adolescent girls and young women in Kenya. J Adolesc Health.

[17] (2015). Fast-tracking combination prevention: towards reducing HIV infections to fewer than 500 000 by 2020. Joint United Nations Programme on HIV/AIDS (UNAIDS).

[18] MTV staying alive foundation. MTV Shuga Down South.

[19] Baker V, Mulwa S, Sarrassat S, Khanyile D, Cousens S, Cawood C, Birdthistle I (2023). 'It is guiding us to protect ourselves': a qualitative investigation into why young people engage with a mass-media HIV education campaign. Cult Health Sex.

[20] Levy J (2015). MTV Shuga - a multi platform communication initiative achieving HIV behaviour change for adolescents in Africa. The Communication Initiative.

[21] Bonell CP, Hargreaves J, Cousens S, Ross D, Hayes R, Petticrew M, Kirkwood BR (2011). Alternatives to randomisation in the evaluation of public health interventions: design challenges and solutions. J Epidemiol Community Health.

[22] Habicht JP, Victora CG, Vaughan JP (1999). Evaluation designs for adequacy, plausibility and probability of public health programme performance and impact. Int J Epidemiol.

[23] Shapiro D, Meekers D, Tambashe B (2003). Exposure to the 'SIDA dans la Cité' AIDS prevention television series in Côte' d'Ivoire, sexual risk behaviour and condom use. AIDS Care.

[24] Usdin S, Scheepers E, Goldstein S, Japhet G (2005). Achieving social change on gender-based violence: a report on the impact evaluation of Soul City's fourth series. Soc Sci Med.

[25] Maclure M, Greenland S (1992). Tests for trend and dose response: misinterpretations and alternatives. Am J Epidemiol.

[26] Schaefer R, Gregson S, Fearon E, Hensen B, Hallett TB, Hargreaves JR (2019). HIV prevention cascades: a unifying framework to replicate the successes of treatment cascades. Lancet HIV.

[27] Shahmanesh M, Mthiyane N, Chimbindi N, Zuma T, Dreyer J, Birdthistle I (2019). P407 ‘MTV shuga’: mass media communication, HSV2 and sexual health in adolescent girls and young women in rural south africa. Sex Transm Infect.

[28] Kyegombe N, Zuma T, Hlongwane S, Nhlenyama M, Chimbindi N, Birdthistle I, Floyd S, Seeley J, Shahmanesh M (2022). A qualitative exploration of the salience of MTV-Shuga, an edutainment programme, and adolescents' engagement with sexual and reproductive health information in rural KwaZulu-Natal, South Africa. Sex Reprod Health Matters.

[29] Baker V, Arnold G, Piot S, Thwala L, Glynn J, Hargreaves J, Birdthistle I (2021). Young adults' responses to an African and US-based COVID-19 edutainment miniseries: real-time qualitative analysis of online social media engagement. JMIR Form Res.

